# Elbow flexion reconstruction after arm-sparing excision for high-grade triton sarcoma: a case report

**DOI:** 10.1186/s13256-020-02384-y

**Published:** 2020-07-04

**Authors:** Elise Lupon, Christine Chevreau, Alexandre Lellouch, Dimitry Gangloff, Thomas Meresse

**Affiliations:** 1 Department of Plastic surgery, University Toulouse III Paul Sabatier, Toulouse, France; 2 Vascularized Composite Allotransplantation Laboratory, Center for Transplantation Sciences, Massachusetts General Hospital, Harvard Medical School, 55 Blossom Street, Boston, MA 02114 USA; 3 Medical Oncology, Comprehensive Cancer Center, Claudius Regaud Institute, Institut Universitaire du Cancer de Toulouse Oncopole, 1, avenue Irène Joliot-Curie, 31059 Toulouse, France; 4 Department of Plastic Surgery, European George Pompidou Hospital, University of Paris, Paris, France; 5 Department of Plastic Surgery, Institut Universitaire du Cancer de Toulouse Oncopole, Institut Claudius Regaud, 1, avenue Irène Joliot-Curie, 31059 Toulouse, France

**Keywords:** Malignant peripheral nerve sheath tumor, Malignant triton tumor, Neurofibrosarcoma, Rhabdomyoblastic differentiation

## Abstract

**Background:**

Soft tissue sarcomas affecting the root of an upper extremity raise the question of limb amputation depending on their location, size, and malignancy. Malignant triton tumors are a rare subtype of neurofibrosarcomas that have been poorly reported in the literature. We report the case of a challenging reconstruction of the upper extremity using a pedicled latissimus dorsal flap.

**Case presentation:**

A 25-year-old Occidental man was referred to our sarcoma unit for the management of a large, high-grade malignant peripheral nerve sheath tumor with no regional or distant extension and very fast progression. He was treated first by concomitant neoadjuvant radiotherapy and chemotherapy. Carcinologic excision was performed “en bloc” including the skin, the tumor, and the flexor muscles of our patient’s elbow. Coverage of the skin defect and elbow flexion restoration were achieved by using a homolateral pedicled musculocutaneous latissimus dorsi flap. Histological analysis showed an R0 resection. The reconstruction process recovered a complete bending of his elbow. He is still in remission at 26 months follow-up.

**Conclusions:**

A malignant triton tumor is a rare, aggressive, and high-grade sarcoma. It was successfully treated and this case report describes an effective treatment modality. Reconstructive surgery, allowing large, complete tumor removal, is indispensable after neoadjuvant chemotherapy and radiotherapy.

## Introduction

Sarcomas are rare malignant tumors associated with a unfavourable prognosis. They can affect all tissues and there are many histological forms. Limbs represent 65% of the locations: 50% at the lower extremity and 15% at the upper extremity [1]. Sarcomas of the root of limbs raise the question of amputation of the affected limb depending on their location, size, and malignancy [2–4]. Malignant peripheral nerve sheath tumors (MPNSTs) are a rare anatomopathological subtype of soft tissue sarcomas (STSs), which account for approximately 2% of cancers; MPNSTs have an incidence estimated at between 4 and 5 cases/100,000 [5] and account for approximately 2% of STSs [6, 7]. In approximately 15% of cases, there are heterotopic elements. MPNSTs with heterotopic elements that are striated muscle fibers are called triton tumors; they are mainly described in patients with neurofibromatosis type 1 (NF1) with aggressive behavior [8]. The therapeutic management modalities of MPNST do not present any specificity and are similar to the recommendations defined for all STSs [9], with the following “pivotal” steps: multidisciplinary team discussion (MTD), histologic diagnostic before any treatment [10], and surgical management consisting of complete excision, with microscopically healthy margins (called R0 excision). There have been very few reports of management modalities for triton tumors.

We present the case of a young man with a rare subtype of MPNST with a rhabdo-myo-chondrosarcomatous contingent who was able to keep his affected limb functional because of neoadjuvant radiochemotherapy and large excision surgery with reconstruction in one step.

## Case presentation

A 25-year-old Occidental man was referred to our sarcoma unit after an inadequate “whoops” surgery excision for a 5.3 cm mass of the biceps brachial muscle of his right dominant upper extremity. A histological analysis revealed a high-grade MPNST sarcoma. He had no significant past medical history. He smoked half a pack of cigarettes a day for 5 years. No case of NF1 had been found in his past family history. A chest computed tomography (CT) scan and positron emission tomography (PET) scan work-up for spread were negative, and a postoperative magnetic resonance imaging (MRI) was performed. The tumor was staged T2N0M0 according to the TNM classification.

Our multidisciplinary staff decided to start a neoadjuvant radio-chemotherapy treatment, which was urgent in view of the aggressivity of the tumor, the incomplete initial surgery, and the macroscopic residue shown on the MRI. Our patient initially refused this treatment. He came back 5 months later with a voluminous painful and fast-growing mass affecting the anterolateral surface of his arm with a radial paralysis. The tumor worsened and was evaluated T3bN0M0 at this time. He finally accepted the treatment.

Chemotherapy with an anthracycline and ifosfamide, that is Adriamycin (doxorubicin) and Holoxan (ifosfamide), and concomitant radiotherapy were administered. More specifically, four cycles of doxorubicin and ifosfamide, including 3 days of treatment every 21 days, were administered. Regarding radiotherapy, our patient received 50.4 Gray in 28 fractions of 1.8 gray each.

**Fig. 1:**
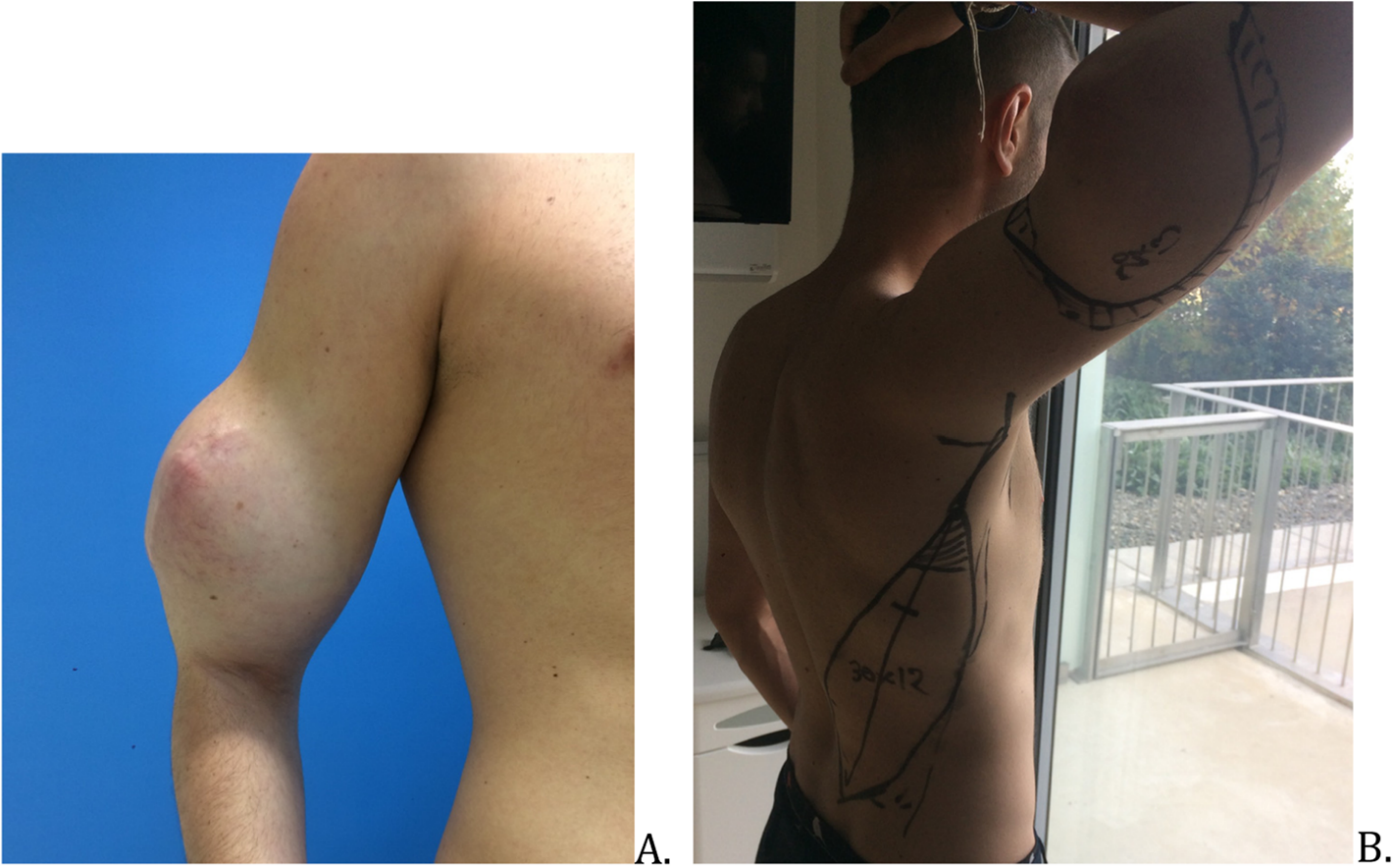
Tumor removal planned with reconstruction by a large dorsal musculocutaneous flap. **a** Tumor in place, front view. **b** Preoperative drawing of the tumor removal and pallet of the large dorsal muscle, dorsal view

**Fig. 2:**
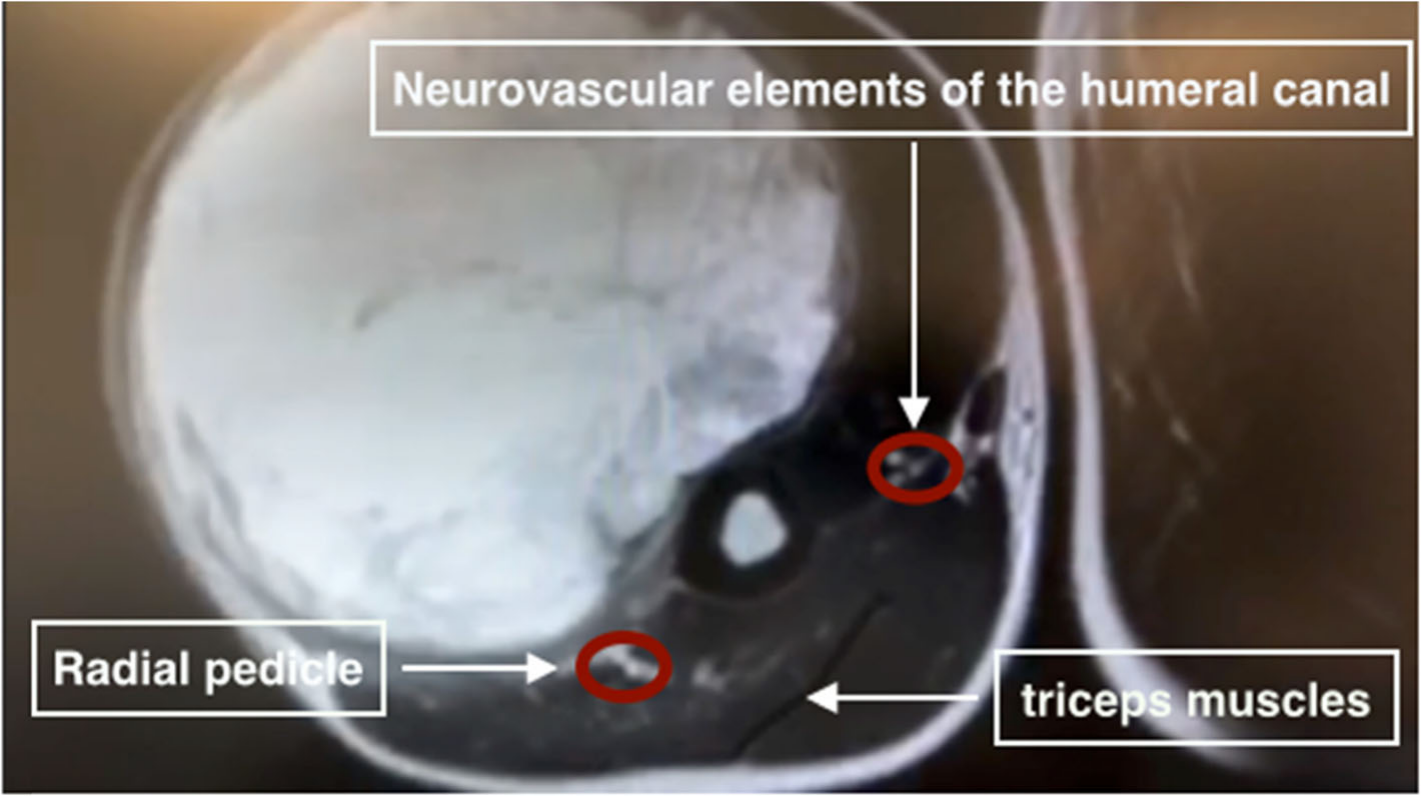
Imaging of the tumor and its relationship to peripheral neurovascular elements

**Fig. 3:**
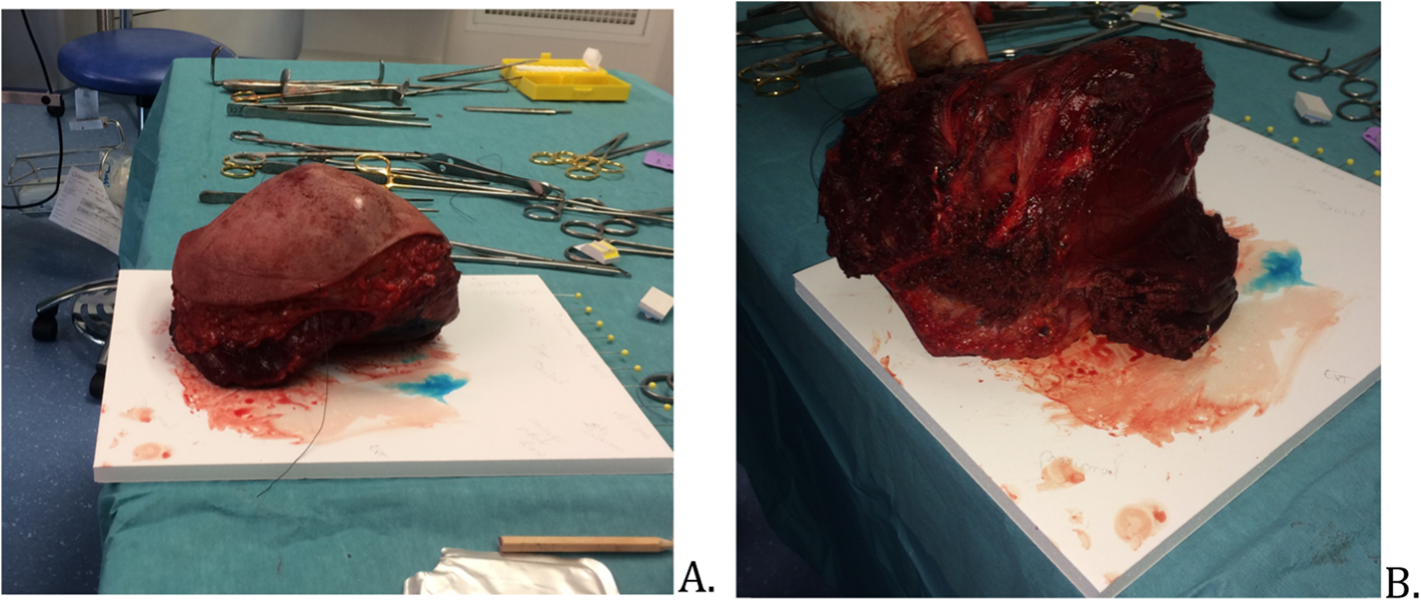
Surgical part enclosed in healthy tissues, with an invisible tumor. **a** External view. **b** Internal view

**Fig. 4:**
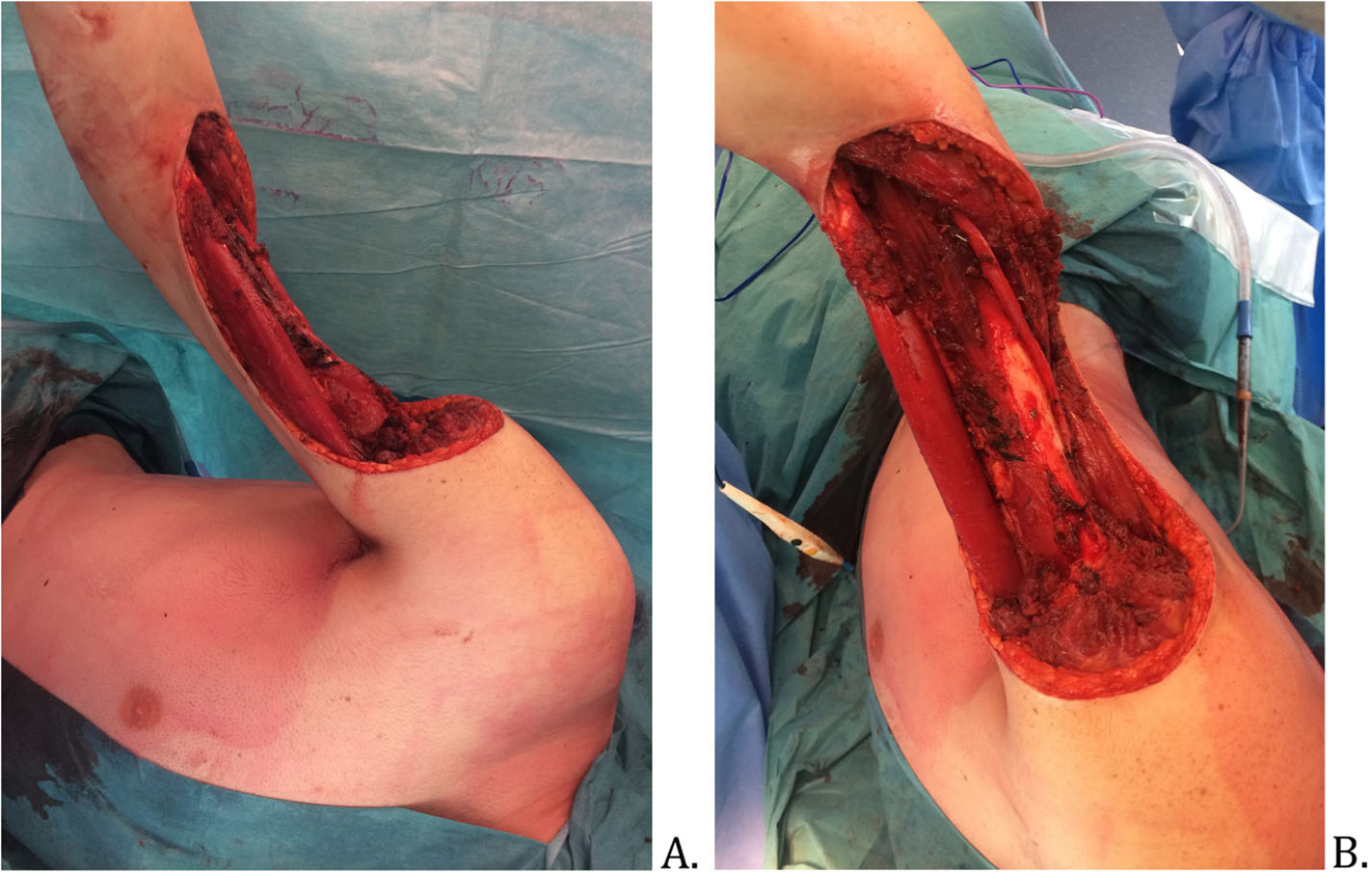
Skin defect after tumor resection. **a** Profile view. **b** Front view

The surgical procedure was planned 6 weeks after the last radiotherapy session (Fig. 1). A MRI showed a tumor with a 15 cm long axis and the different ratios of the tumor to the neurovascular elements were specified (Fig. 2). The surgery was performed in lateral decubitus. Carcinologic excision was performed “en bloc” removing all tissues surrounding the tumor. A macroscopically complete resection was performed, without fragmentation or visualization of the tumor (Fig. 3). The removal of elbow flexor muscles, long head of the biceps muscle, coracobrachialis muscle, anterior brachial muscle, and brachioradial muscle, was necessary. A part of the deltoid muscle and the short head of the triceps were also removed without major consequences to their function. The vascular and nerve pedicles could all be preserved, except the musculocutaneous nerve. The resection was carried out deep down to the bone with removal of the periosteum. Distally, the vessels and nerves were released up to elbow groove and the tendon of the long head of the biceps brachii was preserved. After tumor resection, the tissue defect was extensive (Fig. 4).

The skin coverage and elbow flexion restoration were performed by a large homolateral pedicled latissimus dorsi (LD) flap with a large vertical skin island (30 × 12 cm). The LD muscle was harvested with its distal insertion fascia, on the iliac crest, in order to create a neotendon. There was no detachment of the LD muscle from his humeral tendon. A subcutaneous tunnel was made under the remaining skin of his arm and the flap could go from the back to his arm. Reconstruction of the flexion of his elbow was done suturing the remaining tendon of the biceps brachial muscle to the LD flap neotendon. The donor site was closed with high tension because of lack of laxity in this young patient.

**Fig. 5:**
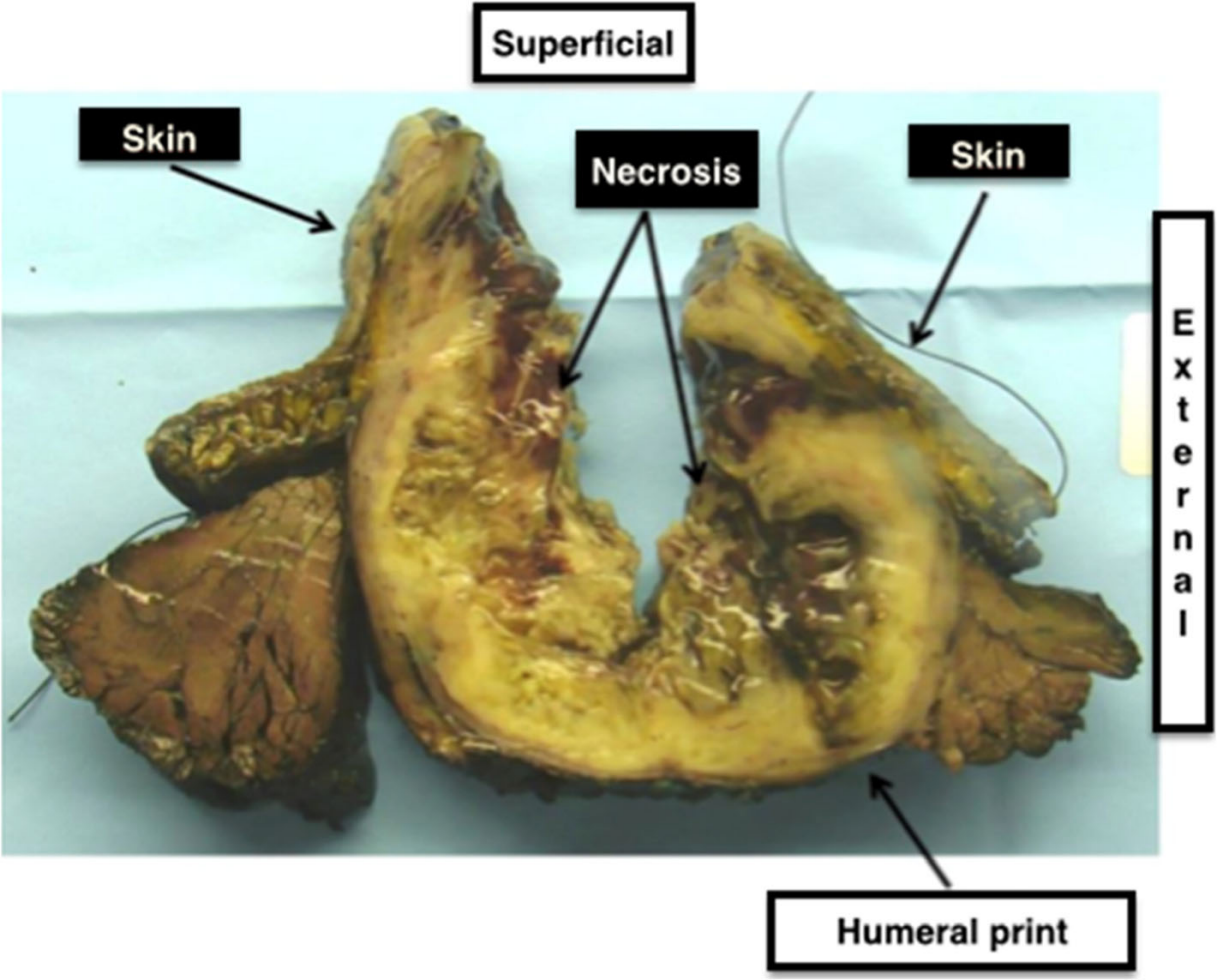
Histological analysis of the tumor

The mass was sent to histology, showing a complete excision of the tumor R0 with a minimum margin of 0.5 mm against the humeral impression including the interposition of the periosteum (Fig. 5), other margins were: 4 mm opposite the impression of the radial nerve, 9 mm laterally, and more than 10 mm in the other directions.

**Fig. 6:**
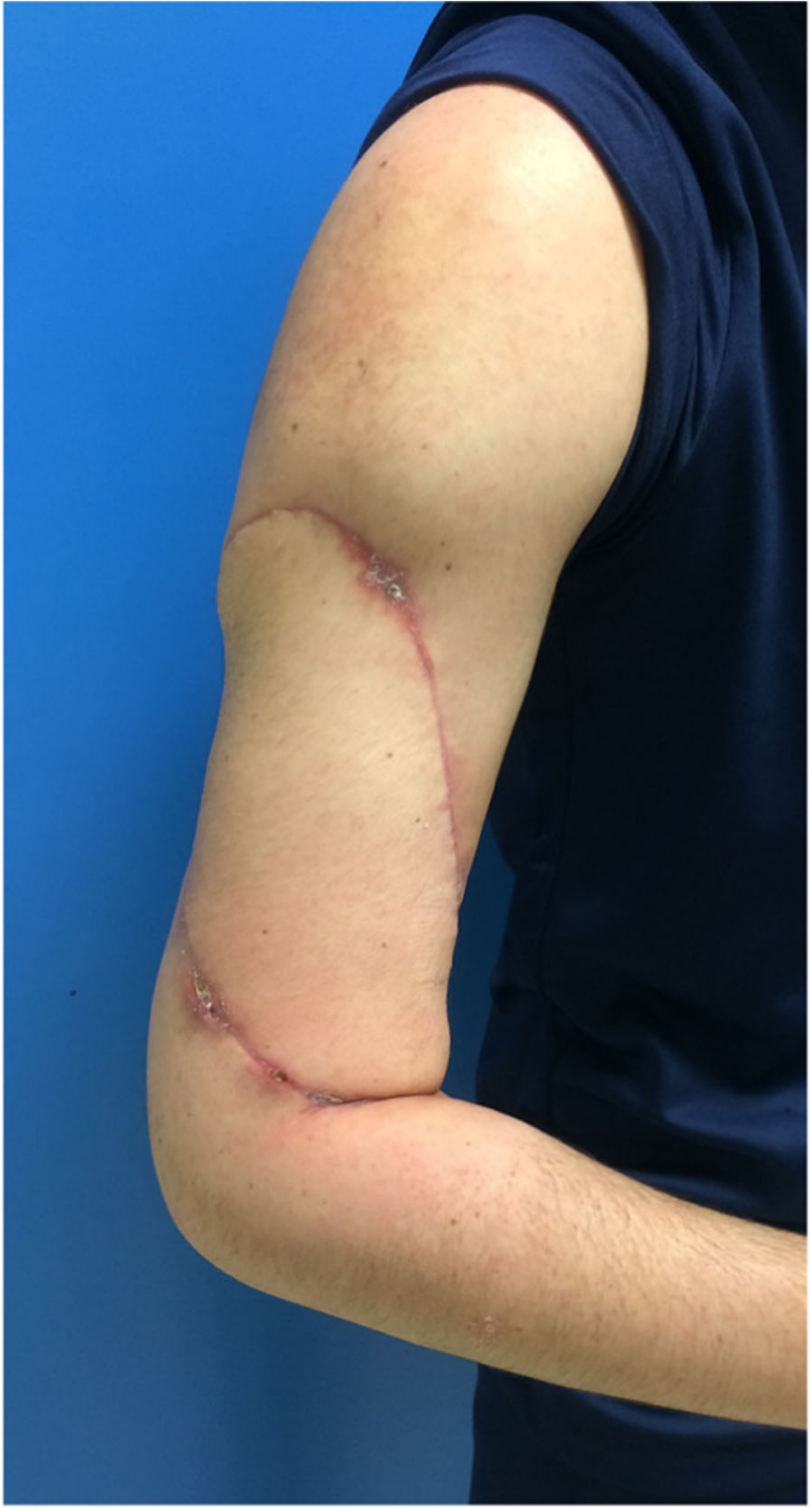
Postoperative flap aspect

This high-grade spindle cell and pleomorphic sarcoma had a dual heterologous component of cartilage and striated muscle type and long bundles of nerve appearance in some areas. This was a rare subtype of sarcoma: a malignant triton tumor (MTT) or MPNST with heterologous chondrosarcomatous and rhabdomyosarcomatous heterologous contingent. There was 50% necrosis and 25% viable tumor cells indicating a partial therapeutic response to chemotherapy. No postoperative complication was noticed (Fig. 6). Our patient was healed at a 3-week postoperative consultation.

Physiotherapy was started at 6 weeks. At 6 months, he was able to get back to work and physical activity. He recovered a full range of motion of the elbow (video 1). The average active bending of the elbow was 140°. At 2-year follow-up, no recurrence was diagnosed (local MRI and thoracic CT scan).


**Additional file 1:Video 1.** Postoperative monitoring.


## Discussion

We report the case of a young man with a MTT. He had a limb-sparing excision with functional reconstruction and kept a full range of motion. We share this case because triton tumors are very rare and few cases are reported.

MTT is a rare subtype of MPNST; it is a neurogenic tumor in which the neurological component induces the production of skeletal muscle [8, 11]. This composite neoplasia was initially described by Masson and Martin in 1938; this tumor is extremely rare, with less than 100 cases reported to date [12]. It mainly manifests itself at the cephalic, cervical, and trunk levels. The diagnosis is based on the presence of malignant rhabdomyoblasts and Schwann cells [13]. The head and neck are the most frequent sites of damage (one-third of lesions), followed by the trunk and lower limbs [14]. Usually seen in people under 35 years of age, the prognosis for MTT is much worse than that of MPNST with an expected 5-year survival rate of 12.5% [15–17]. The sporadic appearance of MTTs in the upper limbs, without NF1 or prior irradiation, is rarely described in the literature. The estimated incidence of MPNST in patients with NF1 is 2 to 5% compared to 0.0001% in the general population and approximately 69% of reported cases of MTT are associated with von Recklinghausen disease [18]. The pathogenesis of sporadic MPNST is poorly known, but the available data suggest different genetic abnormalities from MPNST on NF1, the main one being the independence of NF1 loss in more than half of cases [19]. Cytogenetic studies have revealed some karyotypic changes associated with this tumor. There is a break in 11p15, considered a region of myogenic differentiation. This gene is probably responsible for rhabdomyoblastic differentiation. The amplification of *c*-*myc* oncogene is probably responsible for its aggressive biological behavior [20, 21]. There are still too many errors in initial management, as was the case for our patient, who is nevertheless a crucial and well-documented case [9], which can lead to a significant loss of opportunity for patients [22–25].

There are no specific guidelines for the management of MTTs and therefore the guidelines used are those for STSs. International recommendations have been established to manage such tumors, in an attempt to standardize the therapeutic approach to sarcomas and to get better results [9, 26–28].

A consensus appeared: as soon as a sarcoma tumor is suspected, a thorough imaging assessment must be associated with a biopsy to allow preparation of the surgical procedure in the framework of a multidisciplinary consultation. The excision should take off the whole tumor en bloc. The adjuvant treatment may include radiotherapy and chemotherapy after multidisciplinary consultation.

Thus, the management of MTTs is that of high-grade sarcomas according to the classification of sarcomas by the National Federation of Cancer Control Centers [29]. The only curative treatment for MPNST and most of the prognoses of sarcomas, in the event of a negative extension assessment, are based on broad, complete surgical excision. This corresponds to surgery with a margin of healthy peritumoral tissue, with microscopically healthy removal limits (R0). This surgery must be planned, once the anatomopathological diagnosis has been made, and performed by a surgeon specialized in the management of sarcomas. We had thus scheduled our surgery in a multidisciplinary consultation meeting 6 weeks before the last sessions of the neoadjuvant treatment and the surgical strategy had also been anticipated at the start of treatment at the first surgical consultation. Unplanned resection (whoops surgery) remains a common problem in the management of sarcoma and can seriously compromise the patient’s vital prognosis by increasing morbidity and worsening surgical outcomes [30]. The surgical tumor margin (STM) is the most important measure of sarcoma treatment success, but the definition of the STM has remained a source of controversy. In fact, there is a multitude of literature on sarcoma excision and local recidivism and the margin classifications used vary considerably.

Our resection has been classified R0 according to the Union for International Cancer Control classification. There was an area where the nerve was only separated from the tumor by fat, leaving doubt about R1 excision on histological analysis. This risk, anticipated on imaging and identified during the operation, was accepted because in the worst case it would have corresponded to a programmed R1 resection. In fact, the Toronto Margin Context Classification does not find any significant difference in long-term survival between a programmed near-positive margin excision (R1) and a healthy margin excision (R0) [31].

However, this lack of consistency between and within margin classification systems has been highlighted [32]. We believe that there is no quantified margin to be respected. The main part of this surgery consists in taking with the tumors intact an anatomical unit of interposition (which is often a fascia), as shown by some authors [33, 34]. This concept derives from the work of Enneking *et al.* [35] in which a reactive zone around sarcomas contains tumor cells. This work specifies that resection through this layer is a “marginal” excision, while surgery outside this layer is called “broad.” When an entire compartment is resected, then the resection is considered radical. If the tumor itself is pierced at any stage, then this is considered intralesional excision [36]. In fact, very high levels of local control (94%) can be achieved in STSs with negative margins [37]. Chemotherapy can significantly improve this margin [38, 39]. The indications for adjuvant and neoadjuvant treatments do not present any specificity for MPNST compared to STSs in general. The principle of neoadjuvant treatment is discussed (depending on age, grade, lesioned topography, that is, suprafascial or subfascial plane tumor) in the presence of a disease that cannot be re-secured from the outset, or of excision requiring mutilating surgery. It must be discussed on a case-by-case basis, in a multidisciplinary consultation meeting [7–9]. Currently, there are no recommendations for chemotherapy in MTTs. Our center opted, in a multidisciplinary consultation meeting, to carry out neoadjuvant radiochemotherapy. The objective of this preoperative chemotherapy was to reduce tumor size and optimize the surgical procedure. The tumor decreased by 5 cm and a recovery of radial paralysis was gradually observed after the initiation of chemotherapy and it was, and argued to be, as conservative as possible on the radial nerve which was probably compressed by the tumor rather than invaded. A recent study showed that preoperative neoadjuvant chemotherapy for the treatment of sarcoma significantly improves limb recovery, disease control, and overall survival, and is an effective and safe option for patients with osteosarcoma [40]. We believe that when surgical reconstruction is possible downstream, neo-chemotherapy and neo-radiotherapy are justified and optimal in the conservative treatment of these high-grade STSs. The authors of the few publications concerning MTT have different recommendations for radiochemotherapy and no optimal strategy has been determined [41–43]. In fact, given the rarity of MTTs, no large-scale trials have been conducted to assess the appropriateness of adjuvant therapy. It was pointed out that reports on successfully processed MTT cases are useful in helping to establish an effective treatment modality [41].

With advances in chemotherapy and surgical techniques, the trend in the treatment of sarcomas continues to progress towards limb conservation [4]. When the limb can be conserved, however, there are challenging problems with the coverage of loss of tissues and loss of function that can be caused by tumor ablation. Plastic surgery allows, for the surgery of limb sarcomas, the avoidance of amputation because of a wide, optimal, and uncompromising excision while ensuring the coverage of the loss of substance and rebuilding function. Plastic surgery is therefore today an essential specialty in a sarcoma referral center. In our case, the loss of the anterior muscle compartment would have compromised the possibility of bending the elbow. However, elbow flexion is a vital function in daily life, especially when reaching for the mouth and dressing alone. We chose a coverage and reconstruction of the elbow flexion by a large dorsal musculocutaneous flap because it provides a high strength and an active range of motion. There is little morbidity at the donor site (except for crutch users, patients with paraplegia, and those who practice climbing). However, it should be noted that there was a significant and unsightly enlargement of the back-sampling scar in our patient, due to a direct high-tension closure, because of the need for a huge skin paddle on a young adult skin with very little laxity.

A skin paddle combined with a flap of LD muscle is particularly useful in such cases, as presented here, where there is a defect in the soft tissues of the arm. It is a reliable flap, especially in irradiated areas with a high risk of scarring disorders, which allows the safe coverage of a very large cutaneous defect. The result in terms of flexion recovery is obtained immediately, which allows very early rehabilitation [44]. All other options for coverage by local muscle transfer were not possible due to the size of the area to be covered. Free flaps, which are more difficult to re-innerve than the large pedicled LD flap, were excluded due to the deterioration of the receiving environment through chemotherapy and tissue irradiation.

## Conclusion

MTTs are a rare subtype of high-grade STSs that can affect upper extremities. Reconstructive surgery associated with radiochemotherapy is essential for tumor control, oncologic outcome, and limb function preservation.

## Data Availability

The dataset used and analyzed during the current study is available from the corresponding author on reasonable request.
