# Correction to: Elbow flexion reconstruction after arm-sparing excision for high-grade triton sarcoma: a case report

**DOI:** 10.1186/s13256-020-02477-8

**Published:** 2020-08-07

**Authors:** Elise Lupon, Christine Chevreau, Alexandre Gaston Lellouch, Dimitry Gangloff, Thomas Meresse

**Affiliations:** 1 Department of Plastic surgery, University Toulouse III Paul Sabatier, Toulouse, France; 2 Vascularized Composite Allotransplantation Laboratory, Center for Transplantation Sciences, Massachusetts General Hospital, Harvard Medical School, 55 Blossom Street, Boston, MA 02114 USA; 3 Medical Oncology, Comprehensive Cancer Center, Claudius Regaud Institute, Institut Universitaire du Cancer de Toulouse Oncopole, 1, avenue Irène Joliot-Curie, 31059 Toulouse, France; 4 Department of Plastic Surgery, European George Pompidou Hospital, University of Paris, Paris, France; 5 Department of Plastic Surgery, Institut Universitaire du Cancer de Toulouse Oncopole, Institut Claudius Regaud, 1, avenue Irène Joliot-Curie, 31059 Toulouse, France

**Correction to: J Med Case Reports 14, 103 (2020)**

**https://doi.org/10.1186/s13256-020-02384-y**

Following publication of the original article [1], the authors identified an error in the author name of Alexandre Gaston Lellouch.

The incorrect author name is: Alexandre Lellouch

The correct author name is: Alexandre Gaston Lellouch

The author group has been updated above and the original article [1] has been corrected.
